# The relationship between the error catastrophe, survival of the flattest, and natural selection

**DOI:** 10.1186/1471-2148-11-2

**Published:** 2011-01-04

**Authors:** Héctor Tejero, Arturo Marín, Francisco Montero

**Affiliations:** 1 Departamento de Bioquímica y Biología Molecular I, Universidad Complutense de Madrid, Avd. Complutense s/n, 28040 Madrid, Spain

## Abstract

**Background:**

The quasispecies model is a general model of evolution that is generally applicable to replication up to high mutation rates. It predicts that at a sufficiently high mutation rate, quasispecies with higher mutational robustness can displace quasispecies with higher replicative capacity, a phenomenon called "survival of the flattest". In some fitness landscapes it also predicts the existence of a maximum mutation rate, called the error threshold, beyond which the quasispecies enters into error catastrophe, losing its genetic information. The aim of this paper is to study the relationship between survival of the flattest and the transition to error catastrophe, as well as the connection between these concepts and natural selection.

**Results:**

By means of a very simplified model, we show that the transition to an error catastrophe corresponds to a value of zero for the selective coefficient of the mutant phenotype with respect to the master phenotype, indicating that transition to the error catastrophe is in this case similar to the selection of a more robust species. This correspondence has been confirmed by considering a single-peak landscape in which sequences are grouped with respect to their Hamming distant from the master sequence. When the robustness of a classe is changed by modification of its quality factor, the distribution of the population changes in accordance with the new value of the robustness, although an error catastrophe can be detected at the same values as in the general case. When two quasispecies of different robustness competes with one another, the entry of one of them into error catastrophe causes displacement of the other, because of the greater robustness of the former. Previous works are explicitly reinterpreted in the light of the results obtained in this paper.

**Conclusions:**

The main conclusion of this paper is that the entry into error catastrophe is a specific case of survival of the flattest acting on phenotypes that differ in the trade-off between replicative ability and mutational robustness. In fact, entry into error catastrophe occurs when the mutant phenotype acquires a selective advantage over the master phenotype. As both entry into error catastrophe and survival of the flattest are caused by natural selection when mutation rate is increased, we propose differentiating between them by the level of selection at which natural selection acts. So we propose to consider the transition to error catastrophe as a phenomenon of intra-quasispecies selection, and survival of the flattest as a phenomenon of inter-quasispecies selection.

## 1. Background

The quasispecies model was developed by Eigen in 1971 [1], and has been applied in many different fields, on account of its usefulness as a general evolutionary model for error-prone self-replicative systems. Prebiotic self-replicating molecules [2], RNA viruses [3], cancer cells [4], the immune system [5], etc. have all been modelled as quasispecies. One of the most important implications of the quasispecies theory, of particular relevance because of its originality and possibility for practical applications, is the concept of error catastrophe [6]. In some fitness landscapes, an increase in the mutation rate beyond an error threshold causes the quasispecies to enter into error catastrophe. In the classical model developed by Eigen, several different phenomena can be observed when the population crosses the error threshold. First, the master sequence, i.e. the sequence with the highest replicative ability, is lost [1]. Second, the quasispecies gets delocalized over the whole sequence space [7], in such a way that sequences become uniformly distributed. As this is materially impossible in finite populations, this delocalization should be interpreted as the drift of the population over the sequence space [8]. Finally, several abrupt changes can be observed for different population traits, namely in the mean and the variance of the Hamming distance from the master sequence [9,10], in some collective order parameters such as the consensus sequence [11] or in the so-called ancestral distribution [12,13], etc. Although in the single-peak fitness landscape used by Eigen, all these phenomena take place at the same value of q, this is not necessarily the case for other, more complicated, fitness landscapes [14]. As Hermisson and co-workers [13] have shown, these phenomena are different kinds of what they call "mutation thresholds". These thresholds were defined as "pronounced changes of the equilibrium distribution of some population trait or fitness values within a narrow change of mutation rates" [13]. They identify four different thresholds: a, a "fitness threshold", which is the mean fitness of the population that suffers the pronounced change; b, a "wild-type threshold", which is defined as the loss of the fittest sequence; c, a "degradation threshold", beyond which fitness no longer changes with the mutation rate; and d, a "trait threshold", defined by a pronounced change in a population trait. Moreover, it has been shown that all these thresholds are completely dependent on the fitness and the mutation landscape [13,15]. One problem derived from this fact is that the definitions of the error catastrophe, and of the error threshold, change from paper to paper [13,14,16]. In most papers the error catastrophe is equated to the loss of the master sequence [1,17-19], in other papers it is taken to be the delocalization of the population over the sequence space [20-23], while in others it is defined as a transition to more robust regions of the sequence space [24]. Actually, in some papers two of these definitions are used simultaneously. However, the usual mathematical criterion for defining the error threshold is the loss of master sequence in the absence of back mutation. The differences that are observed for the phenomenology and definition of error catastrophe, and error threshold, can also be observed in the interpretation of both concepts. Error threshold is said to be a critical mutation rate beyond which one of the following phenomena take place: mutation dominates over selection [13,25,26], natural selection ceases to operate [5,13], there is no mutation-selection balance [18,27], or evolutionary adaptation [28] or optimization [22] breaks down.

Furthermore, both the loss of the master sequence and the delocalization of the quasispecies over the sequence space have been related to an information "crisis" or "meltdown" [6,16,26,29,30]. In this sense, the entry into error catastrophe is supposed to establish a maximum limit on the information content that a self-replicative system can maintain at a given mutation rate [20,31,32]. This interpretation has two important practical consequences. In the field of the origin of life, it introduces what is known as "Eigen's paradox" [1,29,33], according to which the first self-replicating molecules would not be long enough, at prebiotic mutation rates, to encode the enzymes, or functions, required to copy the sequences more accurately. The second important consequence is in the field of RNA viruses [16,34], since the possibility of pushing RNA virus replication into error catastrophe by means of mutagenic drugs was the origin of the first "lethal mutagenesis" experiments, i.e. virus extinction through increased mutagenesis, as well as a first explanation for the loss of viral infectivity [35-38].

Another implication of the quasispecies theory that we would like to emphasize is the "survival of the flattest" concept [39-42]. Basically, the "survival of the flattest" effect postulates that at high mutation rates a quasispecies with high reproductive capacity, but low mutational robustness can be displaced by another quasispecies with lower reproductive capacity but higher mutational robustness, that is to say, a greater insensitivity to the deleterious effect of mutations. This implies that mutational robustness may be optimised by natural selection and, consequently, that quasispecies with higher mutational robustness could be selected at high mutation rates. Apart from furthering our understanding of the natural history of populations of self-replicating species, this effect raises the possibility of a resistance mechanism to therapies based on "lethal mutagenesis", which would be distinct from and somehow complementary to drug resistance. However, this point is still a matter of contention [43-45].

Both transition to error catastrophe and survival of the flattest are related to the behaviour of quasispecies at high mutation rates [18,24,25,46]. Some authors have explicitly related both concepts, pointing out that entry into error catastrophe "requires that some genotypes or phenotypes are more sensitive to mutation than others" [18], that it is "an evolutionary phenomenon in which (...) the population evolves to genotypes that are low in fitness but robust to the effects of mutations" [24], or that is a theory that shows that "the evolutionary potential of a phenotype depends on both its fitness relative to alternative phenotypes and its robustness to mutations" [46]. However, to the best of our knowledge, neither an explicit study of this relationship, nor an explicit reinterpretation of its consequences, has as yet been taken.

This brief review of recent theoretical papers on this subject shows that the interpretation and meaning of error catastrophe, and its consequences, is a contentious subject. In this paper we will show that what has been called an entry into error catastrophe should actually be considered to be as a specific case of survival of the flattest. Accordingly, the transition into error catastrophe is caused by natural selection when the selection pressure is an increment in mutation rate. For this reason, an error threshold can only take place in fitness landscapes with flat enough regions. Thus, entry into error catastrophe is caused neither by the dominance of mutation over selection nor because natural selection ceases to operate.

In section 2.1 we show throughout a very simplified model that the entry into error catastrophe is formally equivalent to the survival of the flattest. In section 2.2 we study how changes the population distribution beyond the error threshold when the mutational robustness of some Hamming classes is changed by modifying its quality factor. In section 2.3 the possible relationship between the entry into error catastrophe and the survival of the flattest is considered. Finally, these and other previous results are interpreted in the discussion by considering the transition to error catastrophe as a specific case of intra-quasispecies survival of the flattest.

## 2. Results

### 2.1. A simple model to show that entry into error catastrophe is caused by survival of the flattest

In order to show that the transition to error catastrophe is caused by the selection of a flatter phenotype at high mutation rates, the simplest quasispecies model that displays an error threshold can be used, applying some purely formal modifications. This model has a master phenotype, composed of a single sequence with an amplification factor A_m_, and a mutant phenotype, which combines all the other sequences and has an amplification factor A_k_. The value of the degradation factor, D, is assumed to be the same for all the sequences and, without loss of generality, is set to D = 0. Throughout this section, the classic notation in which q is the quality factor *per digit *will be used, so the probability of correct replication for the master sequence is q^ν^, ν being the sequence length. As the mutant phenotype is composed of all the mutant sequences except the master sequence, back mutation can be neglected as a first approximation. This means that the probability of a mutant sequence producing another mutant sequence is unity. Assuming that the dynamics takes place in a flux reactor [1,2] the following system of differential equations can be derived:

(1) $$\begin{array}{l} \frac{dx_{m}}{dt}=A_{m}q^{\nu }x_{m}-\phi_{0}x_{m}\\ \frac{dx_{k}}{dt}=A_{m}(1-q^{\nu })x_{m}+A_{k}x_{k}-\phi_{0}x_{k} \end{array}$$

where ϕ is the output flux of information carriers. Assuming the constraint of a constant population, N, this results in:

(2) $$\phi_{0}=\frac{A_{m}x_{m}+A_{k}x_{k}}{N}=\bar{E}$$

where $\bar{E}$ is the mean population productivity.

The definition of the error threshold is not a trivial point, as has been discussed under the Introduction. Throughout this paper the error threshold will be considered to be the value of q at which a "fitness threshold" takes place. This kind of threshold can induce the loss of the master type, i.e. a "wild-type threshold", or the delocalization over the whole sequence space, i.e. a "degradation threshold" [13]. This is equivalent to finding the value of q for which a minimum in the difference of the first and second eigenvalues appears in the equivalent linear system obtained, following [2,47]. However, when back mutation is neglected this approach cannot be used, since the matrix of the linear system is not irreducible. Therefore, without losing generality, in this section the error threshold is obtained by equating *x_m _*(*t *→ ∞) to zero, because in the single-peak landscape the fitness threshold and the wildtype threshold coincide. Taking this into account, the system (1) displays an error threshold at:

(3) $$q_{\text{c}}=\sqrt[{\nu }]{\frac{A_{k}}{A_{m}}}$$

The system of differential equations (1) can be expressed in terms of relative fitness and selection coefficients referred to the master phenotype, as usually done in population genetics. A coefficient of selection, *s*, indicates the selective advantage or disadvantage of a given phenotype with respect to another of reference. So, an s value greater than zero implies a selective advantage for a given phenotype with respect to that of the reference, a value less than zero implies a disadvantage, and a value of zero implies neutrality.

To find an explicit relationship between the selection coefficient and other parameters in this case, the system can be reformulated by making a variable change:

(4) $$\begin{array}{l} z_{m}=A_{m}q^{\nu }x_{m}\\ z_{k}=A_{m}q^{\nu }x_{k} \end{array}$$

which is equivalent to dividing the equations of the system (1) by the effective fitness of the master phenotype, i.e. the product of its replicative ability and its mutational robustness: *A_m _q^ν ^*.

As *A_m _q^ν ^*is constant over time, the resulting system is:

(5) $$\begin{array}{l} \frac{dz_{m}}{dt}=z_{m}-\phi_{0}z_{m}\;\\ \frac{dz_{k}}{dt}=\frac{(1-q^{\nu })z_{m}}{q^{\nu }}+\frac{A_{k}}{A_{m}q^{\nu }}z_{k}-\phi_{0}z_{k} \end{array}$$

The selection coefficient can be obtained from this system of differential equations,, and in this case it is a function of the quality factor and the sequence length:

(6) $$s(q,\nu )=\frac{A_{k}}{A_{m}q^{\nu }}-1$$

Substituting s in equation (5) results in:

(7) $$\begin{array}{l} \frac{dz_{m}}{dt}=z_{m}-\phi_{0}z_{m}\\ \frac{dz_{k}}{dt}=\frac{(1-q^{\nu })z_{m}}{q^{\nu }}+(1+s(q))z_{k}-\phi_{0}z_{k} \end{array}$$

Thus, the selection coefficient is the quotient between the effective fitness of the mutant phenotype over that of the master phenotypes, minus one. Actually, the effective fitness of the mutant phenotype should be considered as $A_{k}{\tilde{Q}}_{k}$, where the tilde denotes that the quality factor is considered at a phenotypic level, with ${\tilde{Q}}_{k}=1$, as back mutation is neglected.

Keeping the constraint of constant population, N, the flux term is now:

(8) ϕ0=Zmqν+(1+s(q))zkN

**Figure 1:**
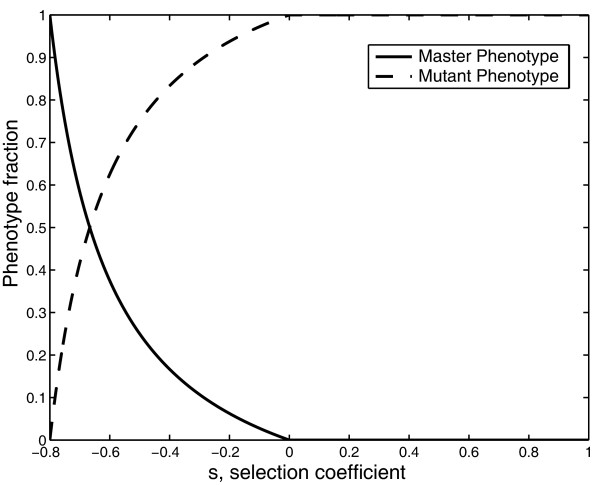
**Phenotype Fraction vs the selection coefficient (s)**. The amplification factors of the master and mutant phenotype are A_m _= 10 and A_k _= 2, respectively. In both cases the degradation factor is D = 1, and the sequence length is ν = 20. The selection coefficient is evaluated as in equation 6.

Taking this into account, and inserting equation (3) into equation (6), it follows that the error threshold is the value of q for which s = 0. That is to say, for q < q_c _the mutant phenotype has a selective advantage over the master phenotype, so s > 0 (Figure 1). It would therefore be incorrect to say that selection prevails before the error threshold, while mutation prevails beyond it. Instead, the master phenotype is selected before the error threshold, while the mutant phenotype is selected beyond this threshold. Thus, entry into error catastrophe is the selection of the mutant phenotype because of the selective advantage provided by its greater mutational robustness. This phenomenon has previously been called survival of the flattest [39-41].

### 2.2. Beyond the error threshold the population evolves to more mutational robust regions of the sequence space

In the previous section, entry into error catastrophe was shown to be the result of natural selection acting on the different effective fitness of the master and the mutant phenotypes. This can also be observed too by using the classical extended model [8] equivalent to the previous one, but in which the mutant sequences are grouped in ν+1 Hamming classes with respect to the master sequence, where ν is the sequence length. In this case back mutation is not neglected, and the system of differential equations takes the general form:

(9) $$\frac{dx_{i}}{dt}=A_{ì}Q_{ii}x_{i}+\sum_{j\neq i}A_{j}Q_{ij}x_{j}-\phi_{0}x_{i}$$

that can be linearized following [2] and then transformed into an eigenvalue-eigenvector problem in the form of:

(10) Wλ=λν

The matrix W is given by *W *= *QA *- *D *, where matrices A and D are diagonal matrices whose elements are the amplification and degradation factors of the different Hamming classes, respectively. Matrix Q is the mutational matrix whose elements Q_ij _determine the probability of obtaining any class i sequence from a class j sequence. The largest eigenvalue, λ, is the mean fitness of the population at the steady state, and its associated right eigenvector v is the population distribution in this state. As this problem is not analytically tractable, even for small sequence lengths, it was solved numerically using MATLAB^®^.

**Figure 2:**
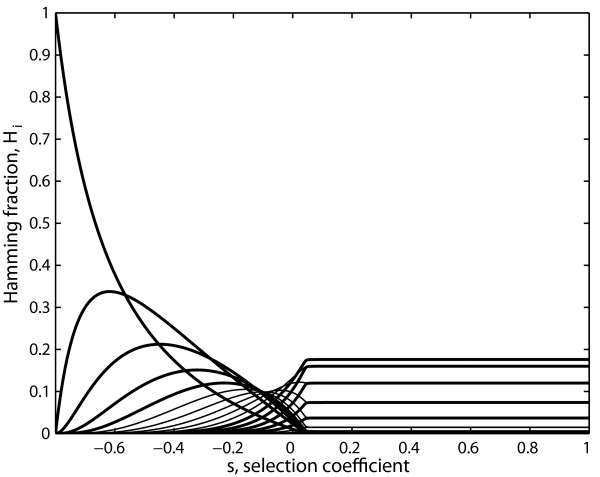
**Hamming class fraction vs the selection coefficient (s)**. The amplification factors of the master sequence, i.e. the zero Hamming class, is A_m _= 10, and that of every other Hamming class H_i _is A_i _= 2. The degradation factor is the same for all the Hamming classes and is D = 1. The sequence length is ν = 20. The selection coefficient is evaluated following equation 6, that is to say considering the mutant phenotype as a whole, not taking into account that it is composed by Hamming classes.

Figure 2 shows that the transitions observed at the error threshold take place when the selection coefficient equals zero. This coefficient has been expressed in a similar way to that of the previous section, and evaluated considering the mutant phenotype as a whole, not taking into account that it is composed by Hamming classes.

Beyond the error threshold, the population is delocalized over the whole sequence space. This delocalization is due to the fact that all the sequences of the mutant phenotype have the same amplification factor and the same quality factor, so natural selection ceases to operate. However, this is an extreme restriction, and no general conclusion should be derived from it. When either the amplification factor or the robustness of some mutant Hamming classes is changed, the population distribution beyond the error threshold departs from the uniform distribution. However, the error threshold is not necessarily modified by these changes.

We will now study how the population distribution beyond the error threshold changes when the robustness of some Hamming classes is modified. As the number of sequences is an intrinsic property of the classification, and depends on the Hamming distance with respect to the master sequence, in order to change the robustness of each Hamming class, we assume that the quality factor *per digit *of each species, q_i_, depends on its sequence, as previously done in [48].

**Figure 3:**
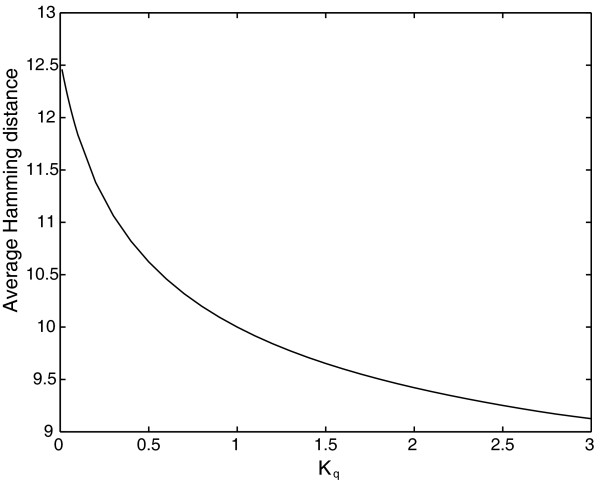
**Average Hamming distance at the error catastrophe when robustness distribution changes**. The quality factor of each sequence depends on the Hamming distance with the master sequence k, according to *q_k _*= *q *if *k *≤ *k_c _*and *q_k _*= 1 - *K_q_*(1 - *q*) if *k *> *k_c _*. In this case *k_c _*= 10. Thus, K_q _is a parameter that modifies the quality factor and the robustness of the Hamming classes. (see text for details). The amplification and degradation factors of the master, and mutant sequences as well as the sequence length are the same as in figure 2.

**Figure 4:**
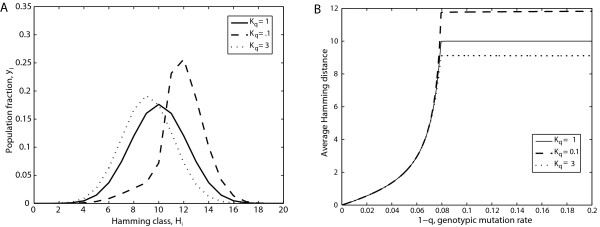
**Changes in population distribution beyond the error threshold for different values of Kq**. Part A. Hamming class distribution beyond the error threshold for different values of Kq. All the distributions have been obtained for q = 0.8. The population for Kq = 1 is the uniform distribution obtained in classical error catastrophe (see text for details). For a greater or lesser value of Kq, the population is displaced to nearest or farther Hamming classes, respectively. Part B. Average Hamming distance as a function of 1-q, for different values of Kq. The changes in Kq does not modify the error threshold, but the average Hamming distance at the error catastrophe is modified as a consequence of the displacement of the population showed in part A of the figure.

In a first approach, the quality factor of each sequence depends on its Hamming distance with respect to the master sequence, so the robustness of each Hamming class can be tuned. By using a truncated mutation landscape the quality factor depends on the Hamming distance in accordance with:

(11) $$\begin{array}{l} q_{k}=q\;\;\text{if}\;\;k\leq k_{c}\\ q_{k}=1-K_{q}(1-q)\;\;\text{if}\;\;k>k_{c} \end{array}$$

where q_k _is the quality factor of the sequences of the Hamming class k, and K_q _a constant we use to change the quality factor of the Hamming classes beyond an arbitrary threshold k_c_. Figure 3 shows the result for k_c _= 10. For K_q _= 1, then q2 = q1, so the quality factor is the same for all the sequences, i.e. the classical model. However, for K_q_ < 1, then q_2 _> q_1_, and the average Hamming distance of the population at error catastrophe increases with respect to the homogeneous case (K_q _= 1), whereas for K_q _>, 1 then q_2 _< q_1_, and the average Hamming distance at error catastrophe decreases with respect to the homogeneous case. However, for any K_q_, the error threshold does not change as, at least for k_c _= 10, the relative effective fitness of the mutant phenotype as a whole with respect to the master phenotype is not affected by the variation in the quality factor. Figure 4 shows the results for the specific case of K_q _= 0.1 and K_q _= 3 compared with K_q _= 1. As we said before, the error threshold does not change. On the other hand, the population distribution at error catastrophe is displaced to greater or lesser Hamming distances, so the average Hamming distance beyond the error threshold increases with respect to the case q1 = q2 (Figure 4b).

### 2.3. Two competing quasispecies with different robustness

In previous sections we showed that the entry intro error catastrophe implies the selection of a flatter phenotype when the population confronts a higher mutational pressure. In this section we will consider the possible relationship between terror catastrophe within a 12 quasispecies and the survival of the flattest between quasispecies. To study this relationship a simplified model of two quasispecies, A and B, similar to those presented in [49-51] to study the survival of the flattest, has been used. In this model, quasispecies A and B compete with each other but there is no mutational flow between them, i.e. a component of a quasispecies is very unlikely to produce a component of the other quasispecies by mutation. Each quasispecies is made up of a master phenotype (m), and a mutant phenotype (k). The four phenotypes have amplification factors A_Am_, A_Ak_, A_Bm _and A_Bk_, in each of which the first subscript specifies the quasispecies and the second the phenotype. The degradation factor, D, is the same for all of them and equal to zero.

The *per digit *genotypic quality factor q is also assumed to be also the same for all of them, but each phenotype must have a different mutational robustness. The phenotypic quality factor of each phenotype i is determined by the expression ${\tilde{Q}}_{i}=\exp[a_{i}(1-q)]$, where a_i _is an arbitrary parameter inversely related to robustness [42,52]. Consequently, the master and mutant phenotypes of quasispecies A have a robustness inversely related to a_Am _and a_Ak_, respectively and, in a similar way, those of quasispecies B have a robustness inversely related to a_Bm _and a_Bk_.

Taking this into account, it is possible to obtain the following system of linear differential equations:

(12) $$\begin{array}{l} \frac{dx_{Am}}{dt}=(A_{Am}{\tilde{Q}}_{Am}-D)x_{Am}+A_{Ak}(1-{\tilde{Q}}_{Ak})x_{Ak}-\phi_{0}x_{Am}\\ \frac{dx_{Ak}}{dt}=A_{Am}(1-{\tilde{Q}}_{Am})x_{Am}+(A_{Ak}{\tilde{Q}}_{Ak}-D)x_{Ak}-\phi_{0}x_{Ak}\\ \frac{dx_{Bm}}{dt}=(A_{Bm}{\tilde{Q}}_{Bm}-D)x_{Bm}+A_{Bk}(1-{\tilde{Q}}_{Bk})x_{Bk}-\phi_{0}x_{Bm}\\ \frac{dx_{Bk}}{dt}=A_{Bm}(1-{\tilde{Q}}_{Bm})x_{Bm}+(A_{Bk}{\tilde{Q}}_{Bk}-D)x_{Bk}-\phi_{0}x_{Bk} \end{array}$$

Assuming a constant population size N, the flux term φ_0 _in these is given by:

(13) $$\phi_{0}=\frac{(A_{Am}-D)x_{Am}+(A_{Ak}-D)x_{Ak}+(A_{Bm}-D)x_{Bm}+(A_{Bk}-D)x_{Bk}}{N}$$

**Figure 5:**
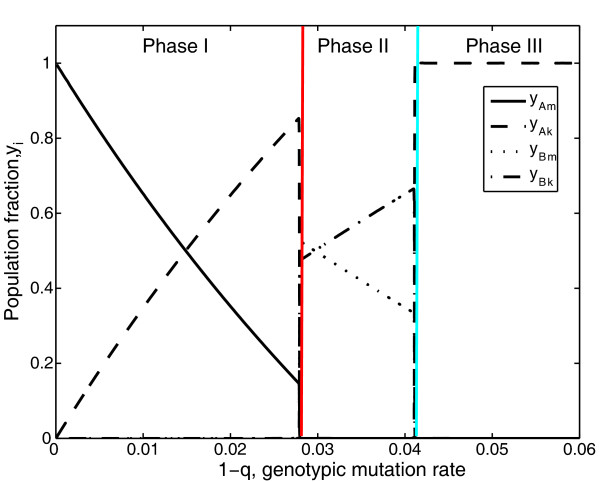
**Population fraction for two competing quasispecies as a function of genotypic mutation rate**. The figure shows the population fraction obtained from equations 12 at the steady state as a function of genotypic mutation rate. Colored lines show the frontiers between the three phases in which alternate quasispecies dominate. In phase I and III, quasispecies A is selected, whereas in phase II, quasispecies B is selected. The amplification factors of quasispecies A are A_Am _= 10 and A_Ak _= 6. Those of quasispecies B are A_Bm _= 8 and A_Bk _= 5. The value of a_i_, which determines the effect of the genotypic mutation rate on the phenotypic mutation rate are a_Am _= 15, a_Ak _= 0, a_Bm _= 7, and a_Bk _= 0.

Figure 5 shows the solution of the model at the steady state, in the absence of back mutation from the mutant to the master phenotype (a_Ak _= 0; a_Bk _= 0). In this figure three regimes, clearly differentiated by two transitions, can be distinguished. Quasispecies A is dominant in the first and the third regime, whereas quasispecies B is dominant in the second. Between these three regimes, two "survival of the flattest"-like transitions take place. The first one at q = 0.9719 and the second one at q = 0.9589 (Figure 5 red and blue line respectively). Of course, in each regime the population distribution of each quasispecies changes as the mutation rate varies. Actually, it is precisely the change in the internal population distribution of quasispecies A and B which modifies the result of the competition between both quasispecies. Although quasispecies A and B are coupled through competition, their internal dynamics is unaffected by that competition. An analysis of steady states allows the error thresholds for both quasispecies to be evaluated. For the specific conditions studied in Figure 5 q_cA _= 0.9659 and q_cB _= 0.9539 are obtained. Therefore, quasispecies A enters into error catastrophe at a value of q which lies between the values of q of the two "survival of the flattest"-like transitions. In fact, if quasispecies A can displace quasispecies B at q = 0.9589 (Figure 5 blue line) it is because quasispecies A is in a regime of error catastrophe, the more robust one, for q ≤ 0.9659. That is to say, the entry of a quasispecies into a regime of error catastrophe allows it to outcompete another quasispecies through a survival of the flattest phenomenon. Quasispecies B enters into error catastrophe for q < q_cB_, but as the robustness of the mutant phenotypes of both quasispecies is the same (a_Ak _= a_Bk _= 0), the mutant phenotype of quasispecies A outcompetes that of quasispecies B as the amplification factor of the former is higher.

## 3. Discussion

### 3.1. The entry into error catastrophe is an specific case of survival of the flattest

The aim of this paper has been to show that the transition into error catastrophe is the result of natural selection acting on the differences in the trade-off between replicative ability and mutational robustness, also called effective fitness. Thus, entry into error catastrophe is a specific case of survival of the flattest. To show this more clearly, a minimal quasispecies model that displays an error threshold was reformulated in section 2.1 in terms of relative effective fitness and selection coefficients. Using this new formulation, we showed that the error threshold is the value of q for which the selection coefficient, s, equals zero (Figure 1). Therefore the selection coefficient is positive beyond the error threshold, which means that beyond that value of q the mutant phenotype has a selective advantage over the master phenotype.

Equation 6 shows that the selection coefficient is the quotient between the effective fitness of the mutant and the master phenotype. As back mutation has been neglected, the probability of going from any mutant sequence to any other sequence of the mutant phenotype is unity, so it does not appear in the numerator.

In section 2.2 we showed with the classical extended model in which sequences are grouped in Hamming classes that the transitions characteristic of an error threshold can be observed when the selection coefficient obtained in the previous section equals zero (Figure 2). This implies that the use of this formal representation for the selection coefficient is still valid for the extended model, when back mutation is taken into account.

Beyond the error threshold, a quasispecies is delocalized over the sequence space in such a way as to produce a uniform population distribution. This is hardly surprising, as the mutant phenotype comprises all but one of the sequences of the sequence space, so it is effectively flat both with respect to the amplification factor and the quality factor. However, the mutational robustness of some mutant sequences can be modified by considering a quality factor that depends on Hamming distance. In this case, a uniform distribution is not obtained beyond the error threshold, and the population evolves in such a way that the regions of the mutant phenotype with a higher quality factor are more populated. That is to say, a population evolves to regions with a greater mutational robustness, as has been shown previously for other similar situations [53,54]. A remarkable result is the decoupling between the value of the error threshold and the delocalization in error catastrophe. Although the population distribution changes when the mutational robustness of the Hamming classes is modified, the error threshold is not altered (Figure 4b). This can be explained by the fact that changing the robustness distribution of the Hamming classes does not modify the effective fitness of the mutant phenotype as a whole, so the error threshold is not affected. As we will discuss below, this is not the case when lethality is introduced.

### 3.2. Revisiting previous results

Error catastrophe and error thresholds have been extensively studied since they were first postulated in 1971 [1]. In this section, some previous results obtained in different papers on the error threshold and error catastrophe will be reinterpreted in terms of natural selection and survival of the flattest.

#### 3.2.1. Revisiting the effect of lethality

We have previously studied the effect of lethality on the error threshold, and shown that an increase in lethality decreases the error threshold, i.e. the quasispecies enters into error catastrophe at greater mutation rates [55]. If we interpret this result as placing an upper limit to the mutation rate that a quasispecies can tolerate before losing its information, or before extinction, this result is, at least, counterintuitive. However, if lethality is considered within the framework of the minimal model presented in section 2.1, i.e. in terms of selection coefficients, the result obtained makes much more sense. The introduction of lethality according to the scheme presented in [55] decreases the effective fitness of the mutant phenotype by both decreasing the number of productive sequences and the mutational robustness of that phenotype. The mutant phenotype therefore becomes less competitive, with the result that the error threshold decreases. This is reflected in the selection coefficient, which would now depend on the quality factor, q, on the sequence length, ν, and on the number of lethal positions in the sequence, n.

(14) $$s(q,n,\nu )=\frac{A_{k}q^{n}}{A_{m}q^{\nu }}-1$$

The same paper studied the changes in the error catastrophe distribution caused by the introduction of lethality. Briefly, the introduction of lethality decreases the average Hamming distance at the error threshold, which subsequently increases linearly with the mutation rate. As a consequence, the uniform distribution is only obtained at q = 0.5. This result is analogous to the result obtained in section × of this paper, in which the effect of the quality factor that depends on the Hamming distance was considered. The introduction of the lethality scheme used in [55] implies that Hamming classes that are further away from the master sequence have more lethal sequences, and therefore a lesser effective fitness: as both robustness and replicative ability decrease. This means that Hamming classes with fewer lethal sequences have a selective advantage, so that the population distribution is displaced to lower average Hamming distances, once again reproducing previous results [53,54]. However, unlike the case when the quality factor depends on the Hamming distance, the introduction of lethality decreases the error threshold, as the fitness of the mutant phenotype as a whole is changed.

#### 3.2.2. Revisiting the effect of neutrality and canalization

Since error threshold is a selective transition to more robust phenotypes when the mutation rate is increased, we will study in this section how error threshold is affected by considering neutrality and/or canalization in the master and mutant phenotypes. For the sake of clarity, canalization is considered to be the existence of different genotypes that produce the same phenotype [56], and neutrality to be the existence of different phenotypes with the same fitness [57].

As far as we aware, the first account of neutrality in the quasispecies theory was given by Eigen and co-workers [58], who introduced a gene segment with no influence in the replicative ability of the self-replicating species. As mutations in this segment do not affect fitness, the segment can vary freely and it is not taken into account in the maximum length allowed by the error threshold. In our opinion, this points to the existing relation between natural selection and the error threshold.

The introduction of canalization in the master phenotype through neutral networks led the introduction of the distinction between genotypic and phenotypic error thresholds [28]. When the master phenotype is composed of more than just one sequence, the genetic information of the master phenotype is lost for any value of mutation rate different from zero, beyond which the population wanders through the master neutral network [28,59]. It is therefore necessary to define a phenotypic error threshold for which the master phenotype is lost, and the population begins a random walk through the phenotype space. However, this stricter definition does not change the considerations made in the previous section, namely, that a phenotypic error catastrophe has no influence at a higher selective-evolutionary level, as almost all those phenotypes have the same or almost the same fitness. In fact, the very idea of the genotypic error threshold reinforces this conclusion, as the loss of the genetic information of the master phenotypes has not critical consequences for their existence or evolutionary capacities.

Finally, it is possible to define a selection coefficient analogous to the one obtained in section × when neutrality is introduced in the master phenotype.

(15) $$s=\frac{A_{k}{\tilde{Q}}_{k}}{A_{m}{\tilde{Q}}_{m}}-1$$

When, following [31], neutrality is introduced by considering a fraction of neutral substitutions, α, in all possible single substitutions, then ${\tilde{Q}}_{m}={\left\{q+(1-q)\alpha \right\}}^{\nu }$, where ν is the sequence length and q the *per digit *quality factor. If, additionally, back-mutation to the master phenotype from every other phenotype is neglected, which means that ${\tilde{Q}}_{k}=1$, the selection coefficient results in:

(16) $$s(q,\lambda ,\nu )=\frac{A_{k}}{A_{m}{\left\{q+(1-q)\alpha \right\}}^{\nu }}-1$$

which is equal to zero at the error threshold [31]:

(17) $$q_{c}=\left\{{\left(\frac{A_{k}}{A_{m}}\right)}^{\frac{1}{\nu }}-\alpha \right\}\frac{1}{1-\alpha }$$

Equation 16 shows that the effect of increasing the neutral network of the master phenotype is to increase its mutational robustness, and therefore the effective fitness of the master phenotype, at higher mutation rates. As a consequence the mutant phenotype outcompetes the master phenotype at a higher mutation rate than in the case with no neutrality, so error threshold decreases [31,59,60].

#### 3.2.3. Revisiting multiple error thresholds

Several papers have described the existence of multiple error thresholds, also known as error cascades, in complex fitness landscapes [18,61]. In point of fact, this phenomenon had probably been observed previously, although it was not identified as such, and was instead identified as either intermediate regions between high replication accuracy regions and error catastrophe [11] or as partial "delocalization transitions" to flatter regions of the sequence space [21].

From the point of view of the survival of the flattest and natural selection, the appearance of multiple error thresholds is just a consequence of the existence of multiple phenotypes with different trade-offs between replicative ability and mutational robustness [18]. As the effective fitness of these different phenotypes can change differently with mutation rate, natural selection can induce several consecutive transitions between them. Actually, error cascades have been previously related with survival of the flattest [20,61].

Finally, the existence of multiple error thresholds shows that delocalization over a given region of the sequence space is essentially dependent on the phenotype's degeneration. Therefore, delocalization can take place over the whole sequence space, or just over some limited regions corresponding to the neutral network of a given phenotype, as in the case of the so-called genotypic error catastrophe commented above.

### 3.3. Error catastrophe, survival of the flattest, and levels of selection

The main purpose of this paper has been to show that the so-called entry into error catastrophe is a specific form of survival of the flattest, that is to say: it is the consequence of natural selection acting in systems of self-replicating species at high mutation rates. As they are essentially equivalent phenomena, and thus have the same cause and features, we propose differentiating between them by considering that they refer to the two different levels of selection that appears in quasispecies models. The first selection level is that of individual self-replicating species, grouped in phenotypes, which, through selection and mutation, determine the population distribution of the quasispecies. The second is a higher level in which some quasispecies can compete with others, through their emerging biological fitness derived from the interaction of their components [26].

Therefore, we propose to use the term "survival of the flattest" to refer to situations in which two quasispecies compete, and there is no mutational coupling between them. This is either because the number of mutations between them implies that the possibility of obtaining one from the other in a reasonable time is negligible [39,62], or because of structural or functional assumptions that are translated into "replicative isolation" in the model [40-42,49]. On the other hand, entry into "error catastrophe" can be regarded as a selective transition resulting from the competition between two or more mutationally coupled phenotypes within the same quasispecies, each one with a different amplification factor and/or mutational robustness, in which a more robust phenotype displaces partially or totally another phenotype with more replicative ability but less robustness.

The study of the system of differential equations presented in section 2.4 shows that the changes produced by natural selection in the internal population structure of a quasispecies, i.e. entry into error catastrophe, can modify the result of natural selection on the immediately higher level of selection, that of competition between quasispecies. In fact, it can be said that entry into error catastrophe induces a survival of the flattest.

### 3.4. ¿Survival of the flattest vs. survival of the fittest?

At a metaphorical level, survival of the flattest and survival of the fittest are often used to denote two completely different concepts [40,41,49,63]. We think that this is confusing, and can even give rise to serious misunderstandings. We do not believe that the term "survival of the flattest" should be discarded, but neither should it be used to signify the opposite of "survival of the fittest". We think it is more accurate to differentiate between "selection for replicative speed" and "selection for mutational robustness" [41] when comparing both processes. This differentiation stresses that natural selection always promotes selection of the fittest. The point is that when the mutation rate is small, the fittest entity is the entity with the greatest replicative ability, but when the mutation rate is high enough, the fittest entity could be one with the greatest mutational robustness. In the latter case, the fittest entity is the fittest because it is the flattest. Thus, mutational robustness should be regarded as another component of biological fitness, at the same level as the replicative ability, expressed in the amplification factor A_i_. Accordingly, mutational robustness may play a key role in determining the effect of natural selection at high mutation rate conditions [40]. This point can be seen more clearly in section 2.1. when, instead of the intrinsic replicative ability, A_i_, the product of the replicative ability and mutational robustness (the effective fitness) are compared, and the transition to error catastrophe arises naturally as a result of natural selection acting on the difference of fitness.

### 3.5. Information crisis? What information crisis?

As briefly commented in the introduction, error catastrophe has been related either to an information crisis [1,6,16,26,29], or to a breakdown of evolutionary adaptation [2,22,28]. In light of the results and interpretations presented in this paper, we think that is quite inaccurate to speak of any kind of crisis beyond the error threshold.

The so-called "information crisis" beyond the error threshold sometimes is associated to the loss of the master sequence [1,26,29], whereas in other cases it is associated to the delocalization over the whole sequence space [2]. When the quasispecies crosses the error threshold, information is said to melt, suffering a phase transition [6,16,26,64]. As Hermisson et al [13] have shown, both phenomena coincide for the single-peak landscape, but not for other landscapes. In any case, neither the delocalization beyond the error threshold nor the loss of the master sequence can be associated to a critical loss of information.

In the first place, as Eigen has shown, natural selection implies displacements in the 'information space', which can take the form of phase transitions [62,64]. A phase transition is a critical phenomenon, but it is critical because of some physico-mathematical characteristics of the transition and not necessarily because of the effects it produces. The error threshold shows a phase transition for collective order parameters such the consensus sequence [11,16], but not, for example, for the average fitness of the population, whose value does not greatly vary at the error threshold, although its dependence on mutation rate does [13]. Secondly, although application of the concept of "information" to biology can be very useful, it does have its limitations. Delocalization beyond the error threshold causes an "information melting", but only at the symbolic level (i.e. the genotype), the one to which the mathematical theory of communication can be applied [65]. However, as all the mutant sequences can be grouped in the same phenotype, and with the same fitness, the symbolic delocalization has no critical consequences at the evolutionary, i.e. biological, level. As a matter of fact, taking the information metaphor further, it could be said that this "symbolic melting" has no consequences at higher informational levels: neither semantic (the meaning, i.e.: the phenotype) nor syntactic (relation between meanings or phenotypes).

Finally, the loss of the master sequence obviously implies the loss of the information contained in it. However, any natural selection process implies changes in the informational characteristics of the population. Obviously, if the result of natural selection is the disappearance of a given phenotype of the population, that implies an information loss, but, at the same time, it implies an increment in biological fitness. Again, no critical consequence can be expected.

A similar reasoning can be applied to the idea of a "breakdown of evolutionary adaptation" [22,28]. As a result of natural selection, entry into error catastrophe does not imply the "breakdown of evolutionary adaptation" but, on the contrary, the adaptation to higher mutation rates by increasing the mutational robustness of the population. If there is no adaptation beyond that point it is because the population has reached the most adapted state allowed by the model.

In the specific case of the single-peak fitness landscape, entry into error catastrophe would be the most extreme case of selection for mutational robustness within a quasispecies. On the one hand, there is a master phenotype which, as it is made up of a single genotype, the master sequence, has null mutational robustness. On the other hand, there is a mutant phenotype which, as it is made up of the rest of the 2^ν ^-1 possible sequences, has a virtually infinite mutational robustness (and thus, a_iK _= 0 in section 2.3). When confronting a mutational pressure, an infinite mutational robustness is probably one of the best adaptations that a system can reach. Although the mean fitness is insensitive to further increases in mutation rate beyond the error threshold, the condition of which Hermisson et al. [13] have termed a "degradation threshold", this insensitivity results from the fact that the populations has reached an infinite robust phenotype, i.e. the fittest phenotype, which means that calling this phenomenon a "complete mutational degradation" is, at the least, misleading.

### 3.6. Error threshold and RNA viruses

The error threshold concept acquired great importance, among other reasons, when the quasispecies concept was applied to RNA viruses [66], as it established a possible new antiviral mechanism which was radically different to those which had been studied before [36]. In fact, the error threshold concept inspired a series of experimental papers which have demonstrated the possibility of extinguishing some RNA viruses using mutagens, in a process called lethal mutagenesis. However, several authors [18,19] have recently called into question the idea that lethal mutagenesis is a result of entry into error catastrophe, correctly pointing out that error catastrophe is a genetic-evolutionary process while extinction is a demographic process [24]. In this paper we have shown that error catastrophe can be regarded as a particular case of natural selection for mutational robustness within a quasispecies. Consequently, that conclusion is reinforced, i.e. entry into error catastrophe cannot explain viral extinction due to increased mutagenesis. The quasispecies model, however useful it has been for the study of RNA viruses [3,67,68], is still only an initial approach to complex intra- and extracellular viral dynamics.

## Conclusions

The main conclusion of this paper is that the entry into error catastrophe is a specific case of survival of the flattest acting on phenotypes which differ in the trade-off between replicative ability and mutational robustness. In fact, the entry into error catastrophe takes place when the mutant phenotype acquires a selective advantage over the master phenotype. Moreover, beyond the error thresholds, changing the quality factor of some sequences modifies the population distribution at the error catastrophe, displacing it towards the flatter regions of the mutant phenotype. However, the value of the error threshold is not altered by these changes in the mutant phenotype as it depends on its effective fitness as a whole. Both neutrality and some lethality schemes increase the effective fitness of the master phenotype with respect to the mutant phenotype, so error threshold decreases. Taking this into account the notion of crisis information beyond error threshold does not make sense.

As both entry into error catastrophe and survival of the flattest are caused by natural selection when mutation rate is increased, we propose differentiating between them by the level of selection at which natural selection act. Thus, we propose to use the term "survival of the flattest" to refer to situations in which two quasispecies compete, and there is no mutational coupling between them; and the term "entry into error catastrophe" as the displacement of a phenotype with high replicative ability but less robustness by another flatter phenotype, when they are mutationally coupled and within the same quasispecies.

## Competing interests

The authors declare that they have no competing interests.

## Authors' contributions

HT conceived the study, participated in its design and drafted the manuscript. HT and AM carried out the numerical and computational studies. FM participated in its design of the study, coordinated it and helped to draft the manuscript. All authors read and approved the final manuscript.
